# Medical Women

**Published:** 1899-12

**Authors:** 


MEDICAL women are slowly but surely making their way in Germany. The recent decision of the Federal Council that women can be admitted to the State examinations in medicine, pharmacy and dentistry, is accepted as a great concession, although there are many difficulties to be overcome before they can fully profit by this privilege. The men students at Halle, where women are admitted on practically the same terms as men, created quite a sensation, not long ago, by formally protesting that they were losing precious opportunities for acquiring knowledge on account of the restraint imposed on the professors by the presence of women at the lectures, mentioning as one among many instances that when an anatomical point was to be indicated on a subject it was indicated through the sheet, instead of with the sheet drawn down. They appealed to the students of all other universities to maintain their rights to lectures, free from the presence of women. The protesting students were severely reprimanded.—*The Journal.*

